# Association of the Infant Gut Microbiome with Temperament at Nine Months of Age: A Michigan Cohort Study

**DOI:** 10.3390/microorganisms12010214

**Published:** 2024-01-20

**Authors:** Tengfei Ma, Sihan Bu, Adannaya C. Nzerem, Nigel Paneth, Jean M. Kerver, Cybil Nicole Cavalieri, Sarah S. Comstock

**Affiliations:** 1 Department of Epidemiology and Biostatistics, College of Human Medicine, Michigan State University, East Lansing, MI 48824, USApaneth@msu.edu (N.P.);; 2 Department of Public Health Sciences, Henry Ford Health, Detroit, MI 48202, USA; 3 Department of Food Science and Human Nutrition, Michigan State University, East Lansing, MI 48824, USA; 4 Department of Pediatrics and Human Development, College of Human Medicine, Michigan State University, East Lansing, MI 48824, USA

**Keywords:** infant, diet, gut microbiota, neurodevelopment, temperament, Bacteroides, microbiome clustering, vitamin D

## Abstract

Though studies in animals and humans link the gut microbiota to brain development and control of behavior, little research has examined this connection in healthy infants. This prospective study could determine associations between infant gut microbiota at 3 months, and infant temperament at 9 months, in a prospective pregnancy cohort (Michigan Archive for Research on Child Health; *n* = 159). Microbiota profiling with 16S rRNA gene sequencing was conducted on fecal samples obtained at 3 months of age. Based on the relative abundance of gut microbiotas, three groups were identified, and each group was characterized by different microbes. Infant temperament outcomes were reported by mothers using the Infant Behavior Questionnaire-Revised Very Short Form at a mean age of 9.4 months. Fully adjusted multivariate linear regression models showed that certain clusters were associated with higher negative emotionality scores, prominently among infants who had poor vitamin D intake. However, no associations were evident between gut microbiota clusters and temperament scales after FDR correction. After using three differential abundance tools, *Firmicutes* was associated with higher positive affect/surgency scores, whereas *Clostridioides* was associated with lower scores. An association between the gut microbiota and early infancy temperament was observed; thus, this study warrants replication, with a particular focus on vitamin D moderation.

## 1. Introduction

The human gastrointestinal tract is the habitat of trillions of microorganisms that are closely associated with many aspects of human health, including physiological functions, metabolism, and immune function [1,2,3]. A critical time for this community of microorganisms, typically referred to as the microbiota, occurs during the first year of life, as that is when the gastrointestinal tract is exposed to a bacteria-rich environment [4], and dietary intake changes dramatically within those 12 months [5]. The gut microbiota not only aids human health physically [6], but it also plays a role in neurodevelopment through the gut–brain axis [7,8]. The gut–brain axis describes the connection whereby the brain and the microbiota communicate with each other through chemical signaling, and these communications may impact physical and mental health [9].

The bidirectionality of the gut–brain axis has been established in animal models [10]. Studies in mice have demonstrated that altered gut microbiota can lead to higher stress responsiveness [11], anxiety-like behaviors, abnormal social behaviors [12], and autism spectrum disorder-like behaviors [13]. Several studies have shown that germ-free (GF) mice, which have no commensal microbiota and an underdeveloped immune system [14,15], display increased motor activity and reduced anxiety compared with specific pathogen-free (SPF) mice with normal gut microbiota [16]. The abnormalities were partially reversed when the gastrointestinal tracts of the germ-free mice were reconstituted using stools from conventionally raised mice [17]. In addition to the behavioral differences, the brains of GF mice displayed various molecular differences, including brain-region-specific changes in the levels of the brain-derived neurotrophic factor, oxytocin, and vasopressin expression [18].

There is mounting evidence to suggest that the gut microbiota plays a critical role in brain development and the control of behavior in humans as well [19,20]. Cross-sectional studies have identified an association between gut microbiome composition and neuropsychiatric outcomes, including autism spectrum disorder (ASD) and depression [21,22,23]. The disturbance of the gut microbiota in early life may possibly lead to adverse mental health outcomes later in life [24]. Children with ASD have been found to have lower abundances of *Coprococcus*, *Prevotella*, and unclassified *Veillonellaceae* compared with neurotypical children [25], and a significant increase in the *Firmicutes/Bacteroides* ratio exists in children with ASD [26]. However, the absence of an association between ASD severity and the gut microbiota composition of children has also been reported [27,28]. In terms of infant physical development, Sordillo et al. reported that infant gut microbiome composition at 3–6 months was associated with fine motor skills, after assessment with the Ages and Stages Questionnaire at 3 years of age [29]. In a longitudinal study of 201 children, Loughman et al. found that the decreased abundance of the genus, *Prevotella*, in fecal samples collected at 12 months of age, was associated with increased behavioral problems at 2 years of age [30]. In a pilot study, Carlson et al. reported associations between the human infant gut microbiome and fear behavior, as well as possible relationships with fear-related brain structures, at 1 year of age [31].

The microbiome is also thought to play a role in behaviors that determine temperament [32], which comprises constitutionally-based individual differences in emotion, motor behaviors, attention, and self-regulation [33,34]. Longitudinal studies have reported that temperament-based characteristics in early life are associated with psychiatric problems, including anxiety, depression, attention deficit hyperactivity disorder (ADHD), and ASD in mid-childhood [35,36,37]. These studies all suggest that the effects of the microbiome in early infancy and toddlerhood play an important role in brain development and behavior. Despite the importance of temperament-based characteristics, few prospective studies have examined the associations between gut microbiome composition and temperament in infancy. Furthermore, previous studies suggested that sex and vitamin D play roles as effect modifiers, with regard to the association between gut microbiota and neurodevelopment [29,38]. Brain development diverges in males and females in response to hormone production. Tamana et al. reported that Bacteroides is associated with enhanced neurodevelopmental scores in a sex-specific manner [38]. Sordillo et al. confirmed the potential of the vitamin D treatment group in terms of modifying microbiome associations using ASQ-3 scores [29]. Thus, we aimed to investigate the association between the fecal microbiota composition at 3 months of age and infant temperament at 9 months of age. In addition, we also aimed to address the potential of the infant’s sex and vitamin D intake in modifying this association.

## 2. Materials and Methods

### 2.1. Study Participants

The study population was drawn from the Michigan Archive for Research on Child Health (MARCH) cohort [39], an ongoing prospective pregnancy cohort enrolling participants in a stratified random sample of prenatal clinics in Michigan’s lower peninsula. The purpose of the MARCH study is to store biological specimens, and other health information that can be used to understand the causes of problems in pregnancy and the health of children, and to contribute to a nationwide study on child health called the Environmental Influences on Child Health Outcomes (ECHO) [40]. Our analysis included mothers who provided infant stool samples with informed consent. During the phone interview, when the indexed infant was nearly 3 months of age, mothers confirmed their interest in participating in this sample collection. Fecal collection kits were sent by mail as described [41,42]. In the infant’s home, feces were scooped from diapers into collection tubes, and tubes were placed into liquid-safe bags with absorbent material; bags were placed into solid shipping boxes and returned to the lab via the United States’ mail service. We excluded fecal samples collected from infants who were over 3 months old. Finally, 159 samples collected from singleton infants were included in the analysis. This study was approved by the institutional review board of Michigan State University.

### 2.2. Data Collection

At approximately nine months of age, the Infant Behavior Questionnaire-Revised Very Short Form (IBQ-RVSF) was administered to mothers via phone interview to assess their infant’s temperament [43]. In an attempt to address the potential directionality of the relationship between the gut microbiota and infant temperament, we chose to measure infant microbiota at an early point in infancy and temperament later in infancy. The Infant Behavior Questionnaire is a widely used measure that parents use to report infant temperament, and it was first introduced by Rothbart in 1981 [44,45]. The IBQ-RVSF consists of 37 items, measuring three scales of infant temperament, including positive affect/surgency (PAS, 13 items), negative emotionality (NEG, 12 items), and orienting/regulatory capacity (ORC, 12 items) [43]. Each item asked caregivers to report how often their babies engaged in a particular behavior over the previous seven days. The items were rated on a scale ranging from 1 (never) to 7 (always), and then, they were averaged within each of the three scales. Higher scores on each scale indicate that the infant displays more of the measured temperament characteristic. PAS is characterized by high scores on items related to approach, vocal reactivity, high intensity pleasure, smiling and laughter, activity level, and perceptual sensitivity. NEG is analogous to the personality trait of neuroticism, and is characterized by positive readings on sadness, distress to limitations, and fear, as well as negative readings on falling reactivity. ORC is characterized by duration of orientation, low intensity pleasure, cuddliness, and soothability. NEG thus differs from the other two scales in that a high score represents a negative outcome. Studies have demonstrated IBQ-RVSF has adequate internal consistency, test–retest reliability, and interrater agreement between mothers and fathers [43].

In addition to the IBQ-RVSF, multiple questionnaires were administered to mothers from the first prenatal visit until they were 9 months postpartum to collect socioeconomic and other demographic information. Detailed information about the mother, including maternal age, education level, maternal race, pre-pregnancy weight, and height were collected from the maternal survey during the first prenatal visit. Detailed information on the neonate, including sex, date of birth, birth weight, and gestational age, was abstracted from the birth certificate.

### 2.3. Fecal Microbiota Analysis

The fecal samples were aliquoted into sterile tubes and stored at −80 °C, once received in the lab. DNA was extracted by following a modified version of the Human Microbiome Project’s protocol [46]. Barcoded primers were used to amplify the V4 region of the 16S rRNA gene region. The resulting 16S rRNA libraries were sequenced using 250 base pair Illumina MiSeq with V2 chemistry at the MSU genomics core. After trimming, clean sequences were analyzed using the QIIME2 (2021.2 version) pipeline [47]. QIIME2’s DADA2 plugin was used to process the demultiplexed sequences and generate the amplicon sequence variants (ASV) table [48]. ASVs were assigned in a taxonomic manner with the QIIME2 feature–classifier plugin, using the Silva 132 database at a similarity threshold of 99% (for 16S data) [49,50]. Samples were rarefied to 6000 sequencing reads per sample, and taxa present in less than one sample were excluded, leaving 159 stool samples with 6905 unique ASVs. ASVs were summarized at the genus taxonomic level.

### 2.4. Statistical Analysis

All the analyses were performed using R software (version 4.0). The Shannon index, which represents microbial richness and evenness, and the Chao 1 index, which represents microbial richness, were calculated using the R package “vegan” [51]. Associations between the Shannon index and Chao1 index regarding temperament scales were assessed using multivariate linear regression models, which were adjusted in accordance with maternal age, maternal education, maternal race, maternal pre-pregnancy BMI, gestational age, birth weight, infant sex, and infant age at IBQ-RVSF collection. Dirichlet multinomial mixture (DMM) clustering is an unsupervised Bayesian clustering method used to identify clusters of microbial community data [52,53]. We performed DMM, as previously described, using the R package, “DirichletMultinomial” [54,55]. The best fitting DMM model was determined using the Laplace approximation. The differences between alpha diversity and relative abundance of taxa in DMM clusters were tested using Kruskal–Wallis test with the Dunn test for post hoc analysis. Multiple comparisons were adjusted for false discovery rate (FDR) correction using the Benjamini–Hochberg procedure [56]. We performed Principal Coordinates Analysis (PCoA) and Permutational Multivariate Analysis of Variance (PERMANOVA) based on the Bray–Curtis dissimilarity using the “vegan” package to compare microbial community structures in DMM clusters. We used multivariate linear regression models to determine the association between DMM clusters and infant temperament scales, adjusting for maternal age, maternal education, maternal race, maternal pre-pregnancy BMI, gestational age, birth weight, infant sex, and infant age at IBQ-RVSF collection.

We conducted analyses to address the potential for infant vitamin D intake and infant sex to modify gut microbiome associations with temperament. In these analyses, data was stratified by infant vitamin D intake or sex to determine if detected associations between the gut microbiome and temperament were consistent across strata. In our study, infants who were fed with formula or vitamin D supplements were considered as having good vitamin D intake; otherwise, they were considered as having poor vitamin D intake. We utilized DESeq2 [57], ANCOM-BC [58], and MaAslin2 [59] to identify associations between temperament and individual microbiome abundance. All three differential abundance (DA) tools were adjusted using the same covariates. A previous study showed that DESeq2 has a higher false positive rate when rarefied data were used, so we used un-rarefied data for the DESeq2 test [60]. Using these three tools, we focused on obtaining robust, and therefore likely reproducible, results. DESeq2 and MaAslin2 adjusted *p*-values with the use of the Benjamini–Hochberg false discovery rate (FDR) procedure [59], whereas ANCOM-BC used the Holm–Bonferroni Method [61].

## 3. Results

### 3.1. Study Population

One-hundred and fifty-nine participants were included in the final analysis (male = 52.8%, female = 47.2%). Descriptive statistics for the maternal and infant factors, using the three scales measuring infant temperament are displayed in Table 1. More than half of the mothers (57.9%) had earned a college degree, and 80.3% of the participants were white. The mean (SD) age when temperament measurement occurred was 9.4 (SD: 0.7) months, and the median was 9.2 (range: 8.8–13.3) months. White mothers reported significantly lower scores for their infants on all three scales than black mothers. Male infants had significantly higher PAS scores than female infants (*p*-value < 0.001), but had similar scores for the other two scales. Higher maternal age was significantly associated with lower PAS scores (coefficient = −0.03, *p*-value = 0.006) and lower NEG scores (coefficient = −0.04, *p*-value = 0.009).

### 3.2. Alpha Diversity and Temperament Scores

Table 2 presents the association between the alpha diversity of fecal samples and infant temperament scores. A higher Shannon index tended to be associated with higher positive affect/surgency scores (coefficient = 0.11, *p*-value = 0.27) and lower negative emotionality scores (coefficient = −0.18, *p*-value = 0.24), but the associations were not statistically significant. We also did not observe any significant associations between the Chao 1 index and temperament scores (Table 2). No measures of gastrointestinal bacterial alpha diversity were significantly associated with any of the three.

### 3.3. Cluster Analysis

**Figure 1:**
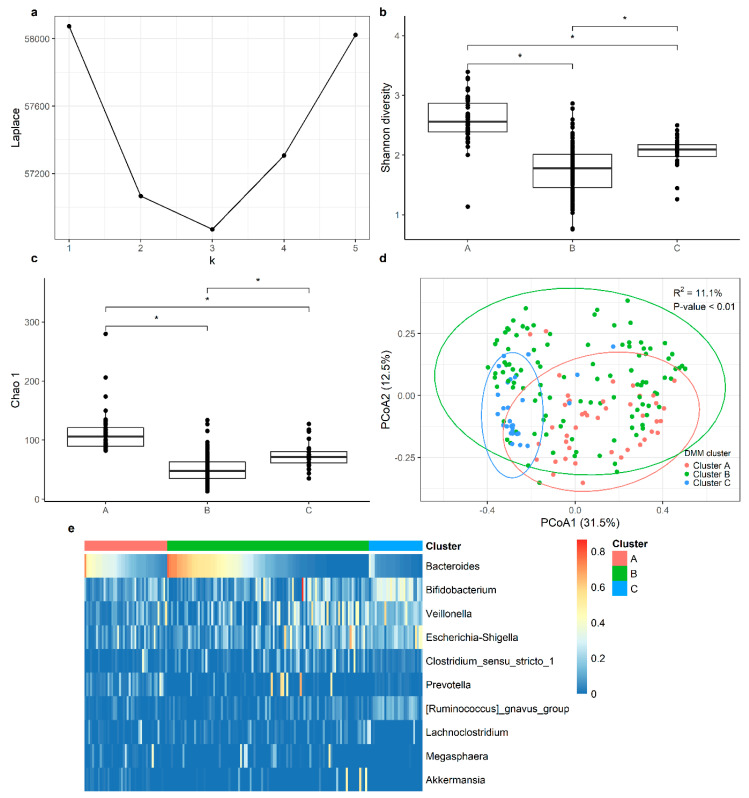
Dirichlet multinomial mixture clustering identified three optimal clusters from 159 fecal samples. (**a**) The number of clusters (k = 3) was chosen by selecting the minimal Laplace approximation, as per the negative log model’s evidence. (**b**,**c**) Boxplot of the alpha diversity (Shannon and Chao 1) distributed between the three clusters. Group differences were tested using Wilcoxon signed-rank test, and *p*-values were adjusted for multiple testing using Bonferroni. The adjusted *p*-value < 0.05 was labeled as *, and the adjusted *p*-value ≥ 0.05 was labeled as NS. (**d**) Principal component analysis (PCoA) ordinations of variation, based on the Bray–Curtis distance matrix. R^2^ and the *p*-value were calculated using the univariate PERMANOVA test. (**e**) Heatmap of relative abundance of the top 10 genera in the three clusters. Within the clusters, samples were ordered in accordance with the relative abundance of the genus, *Bacteroides*.

**Figure 2:**
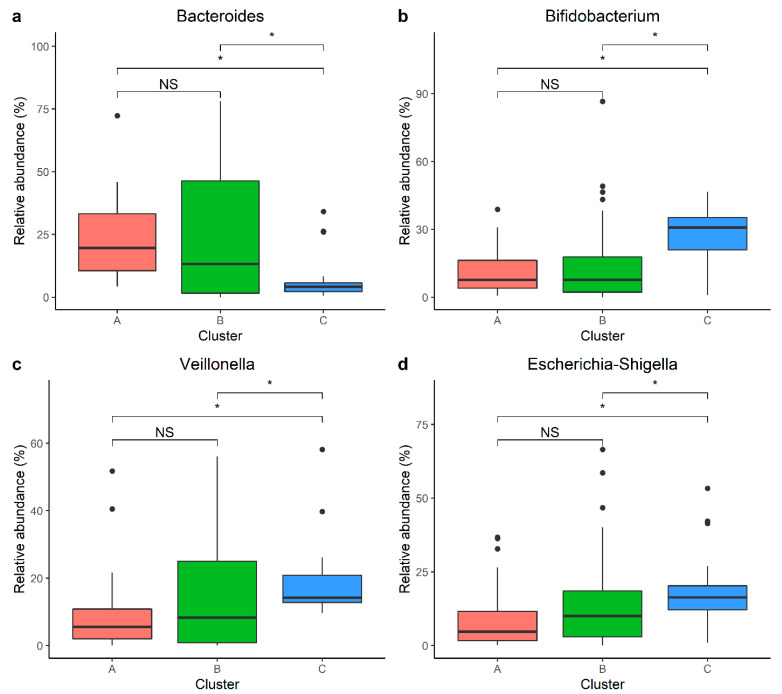
Relative abundance of the top four genera that contributed to the clusters; each cluster is shown. Group differences were tested using the Wilcoxon signed–rank test. *p*-values were adjusted for multiple testing using Bonferroni. The FDR adjusted *p*-value < 0.05 was labeled as *, and the adjusted *p*-value ≥ 0.05 was labeled as NS. (**a**) Boxplot of the relative abundance of *Bacteroides* in each cluster. (**b**) Boxplot of the relative abundance of *Bifidobacterium* in each cluster. (**c**) Boxplot of the relative abundance of *Veillonella* in each cluster. (**d**) Boxplot of the relative abundance of *Escherichia-Shigella* in each cluster.

We employed Dirichlet multinomial mixture (DMM) modeling to assign each participant’s gastrointestinal bacterial communities into a cluster. Using the minimum Laplace approximation, we identified three optimal clusters (Figure 1a). Clusters A, B, and C accounted for 22%, 61%, and 17% of the total samples, respectively. Significantly lower Shannon and Chao 1 indices were observed in cluster B, compared with clusters A and C (Figure 1b,c). Furthermore, the diversity in Shannon and Chao 1 was lower in C than A (Figure 1b,c). In addition, beta diversity was significantly different between the three clusters, based on the Bray–Curtis distance matrix (univariate PERMANOVA: R^2^ = 11.1%, *p*-value = 0.001, Figure 1d). Thus, these results revealed broad bacterial community differences across the three clusters. The heatmap (Figure 1e) shows the relative abundance of the top ten most abundant genera within each cluster. Samples in each cluster were ordered in accordance with the relative abundance of *Bacteroides*. *Bacteroides*, *Bifidobacterium*, *Veillonella*, and *Escherichia-Shigella* were the four genera that the clustering was dependent upon (Figure 2). Samples in cluster A exhibited a higher relative abundance of *Bacteroides* than samples in cluster C (FDR adjusted *p*-value = 8.4 × 10^−10^), but a similar abundance of *Bacteroides* was found in samples in cluster B (FDR adjusted *p*-value = 0.36). Cluster C was characterized by the highest relative abundance of *Bifidobacterium*, *Veillonella*, and *Escherichia-Shigella*, compared with clusters A and B.

To investigate the potential associations between these clusters and infant temperament scores, we applied linear regression models, adjusted by covariates, including the following: maternal age, maternal education, maternal race, maternal pre-pregnancy BMI, gestational age, birth weight, infant sex, and infant age at IBQ-RVSF collection (Table 3). The results of the univariate models demonstrated that cluster A was significantly associated with a higher PAS score (coefficient = 0.41, *p*-value = 0.01, q-value = 0.03), and a higher NEG score (coefficient = 0.57, *p*-value = 0.02, q-value = 0.03), compared with cluster C, after adjustment for multiple testing. In the multivariate linear regression models, both clusters A and B were associated with a higher NEG score compared with cluster C. However, after adjusting for multiple testing, no cluster retained statistically significantly associations with either temperament scale (cluster A: coefficient = 0.55, *p*-value = 0.03, q-value = 0.09; cluster B: coefficient = 0.44, *p*-value = 0.04, q-value = 0.12).

A contingency analysis assessing a possible interaction in the relationship between microbiota and temperament due to vitamin D intake was conducted. An infant was considered to have taken vitamin D if the caregiver reported that the infant consumed infant formula and/or had been given a vitamin D supplement in the 24 h preceding fecal sample collection. Among infants who had not taken additional vitamin D, cluster A infants had significantly higher NEG scores than cluster C infants (coefficient = 0.94, *p*-value = 0.02, q-value = 0.06) before adjustment for multiple testing (Table A1). By contrast, in infants who received additional vitamin D, cluster A infants did not have significantly higher NEG scores, and the relationship was explained by a smaller coefficient (coefficient = 0.59, *p*-value = 0.11, q-value = 0.26). Thus, the association between the gut microbiota cluster and temperament could be attenuated by vitamin D intake. When stratifying in accordance with infant sex, male infants in clusters A and B had significantly higher NEG scores than male infants in cluster C (cluster A: coefficient = 0.92, *p*-value = 0.008, q-value = 0.02; cluster B: coefficient = 0.86, *p*-value = 0.005, q-value = 0.02), whereas no significant association was observed for female infants (Table A2).

### 3.4. Individual Taxa Analysis

We next measured the association between individual taxa at the genus level and infant temperament scales using three differential abundance (DA) tools. The significance cut-offs for DESeq2, ANCOM-BC, and MaAslin were set at 0.001, 0.25, and 0.25, respectively. We displayed both the q-value (Table 4) and coefficient (Table A3) for all three DA tools, for the analysis of the associations between gut microbiota taxa at the genus level and PAS. Infants with higher PAS scores had a significantly higher relative abundance of Firmicutes, including the *Christensenellaceae R-7 group*, *Oscillospiraceae* UCG-002, and *Subdoligranulum*, and a lower relative abundance of *Clostridioides.* These associations were robust for all three DA tests. 

## 4. Discussion

Accumulating evidence from animal and human studies suggests that the gut microbiota plays a role in neurodevelopment during the early, critical time of infancy. In the current study, we focused on the three scales of infant temperament, as follows: positive affect/surgency, negative emotionality, and orienting/regulatory capacity. We observed an association between infant gut microbiota composition and temperament using both clusters and individual taxa. However, some of the relationships were not robust for statistical adjustment using covariates and multiple comparisons.

### 4.1. Alpha Diversity

No significant associations were observed between alpha diversity and temperament scales. This finding is consistent with some results of studies from longitudinal and cross-section studies. Fox et al. investigated the relationship between gut microbiota at each age group (1–3 weeks, 2, 6, and 12 months) and Infant Behavior Questionnaire-Revised scores at 12 months of age [57]. No temperament scales measuring infants at age 12 months demonstrated a significant association between the alpha diversity measures at each age. Similarly, Kelsey et al. demonstrated that neither alpha diversity nor richness was associated with any of the temperament scales in a cross-sectional study [62].

### 4.2. Cluster Analysis

We identified three clusters based on the DMM method. Cluster A was characterized by a higher abundance of *Bacteroides*; cluster C was characterized by a higher abundance of *Bifidobacterium*, *Veillonella*, and *Escherichia-Shigella*; and cluster B exhibited results that fell between those of the other two clusters. Clusters A and B exhibited stronger associations with higher negative emotionality than cluster C, before adjustment for multiple comparisons. Negative emotionality, which is defined as the tendency to experience negative emotions such as anger and fear [63], has often been linked with internalizing and externalizing problems [64,65]. Previous work by Aatsinki et al. also showed the same trend with regard to the relationship between the Bifidobacterium-dominated/Bacteroides-dominated cluster and negative emotionality, though it was not statistically significant [66]. In addition, two studies in early infancy observed the association between the *Bacteroides*-dominant gut microbiota community and poor fine motor skills, which further supports the adverse effect of *Bacteroides*-dominated microbiome composition on infant neurodevelopment [29,55].

In a contingency analysis, we observed that infant vitamin D intake modified the association between microbiota clusters and negative emotionality. The association between cluster A and NEG was only identified among infants who were not consuming vitamin D supplements, suggesting that infant vitamin D intake may protect against the adverse effects of the gut microbiome-associated increments on infant negative emotionality. Our results support those of Sordillo et al.; in the Sordillo study, participants who received prenatal vitamin D, and who exhibited the *Veillonella*-dominated gut microbiota community, were found to have improved communication scores, whereas no association between the *Veillonella*-dominated community and communication scores was observed in the control group [29]. A possible explanation is that vitamin D and vitamin D receptor (VDR) levels may affect the gut microbiota–brain axis by modifying intestinal innate immunity [67]. Numerous studies have shown that vitamin D and VDR have important effects on cells within the immune system [68], and the immune system may continue to interact with the microbiota–brain axis via circulating cytokines [69]. Moreover, our results also suggested that the association between gut microbiota clusters and negative emotionality was driven by male infants. Thus, the gut–brain axis of males appears to be more susceptible to the effects of the gut microbiota, a conclusion also noted by Jašarević et al. [69] and Tamana et al. who showed that the association between *Bacteroides*-dominant gut microbiota and advanced cognitive or language development was only observed in male infants aged two [38]. As males and females exhibit differences in terms of the nutritional and energetic demands of growth, development, and reproduction [70], sex may play an important role in the gut microbiome–brain axis. Hence, our study contributes to the growing evidence that gut microbial composition influences human neurodevelopmental outcomes in a sex-dependent manner.

### 4.3. Individual Taxa

In MARCH infants, several taxa in the gut microbiota were significantly associated with infant temperament outcomes. Of note, our results agree with the previous literature, which suggests that the genus *Clostridioides* in the infant’s gut is associated with adverse neurodevelopment outcomes [71]. *Clostridioides* is an important predictor of infant temperament, as a similar study also reports a significant negative association between *Clostridioides* at age 2.5 months and the regulation scale at age 6 months [66]. *Clostridioides difficile*, which can cause severe diarrhea and colitis, is the most common *Clostridioides* species in infants [72]. In animals, *C. difficile* can produce Propionic Acid, a short-chain fatty acid that can introduce ASD-related symptoms [73,74]. Future research should further examine the specific strain of *Clostridioides* in the infant’s gut to confirm the association between *Clostridioides difficile*, and infant temperament. Our results did not identify any significant associations between *Bifidobacterium* and positive affect/surgency, which differs from other studies. Both Fox et al. and Aatsinki et al. reported that *Bifidobacterium* abundance in early infancy was positively associated with surgency/extraversion [57,66]. *Bifidobacterium* is typically considered to be beneficial for infant neurodevelopment, demonstrating benefits for gut epithelium integrity and function, as well as gastrointestinal motility [75]. A larger sample size may be needed to replicate the previous studies.

### 4.4. Strengths and Limitations

Our current study has a number of strengths. We demonstrated prospective associations between the infant gut microbiota and temperament in a longitudinal pregnancy cohort study. The associations between microbiome clusters and negative emotionality are robust to adjust for covariates. Our analysis not only demonstrated the effect of gut microbiota composition on infant temperament, but it also highlighted the importance of individual taxa. By using three different popular DA tools, we demonstrated that some results were robust, which was due to the choice of the DA tool. However, our study is not free of limitations. First, we adjusted education and maternal race as covariates in our analysis, but the large proportion of white and college graduate participants could still bias the results. Second, although the infant gut microbiota rapidly matures over the first year of life, we had access to just one fecal sample from a single point in time. A single sample cannot fully represent the temporal development of an infant’s gut microbiome, which may be critical to neurodevelopment in early life. Third, the use of the IBQ-RVSF has been shown to not fully represent all aspects of an infant’s temperament, such as soothability and cuddliness. Fourth, 16S rRNA sequencing can only provide limited strain-level information. Previous studies demonstrate the biological importance of the different metabolic capabilities of specific bacterial strains on human health [76]. Thus, shotgun metagenomic sequencing and the metabolomic analysis of stool samples are needed for future studies.

## 5. Conclusions

The current study provides evidence that infant gut microbiota composition in early infancy is associated with temperament in later life, whereas this association is potentially attenuated by infant sex and vitamin D intake. Our results support the growing evidence that alteration of the gut-microbiota–brain axis could lead to changes in behavior or cognition in early life.

## Figures and Tables

**Table 1:** Infant temperament scores concerning the characteristics of mothers and infants.

Scale		Positive Affect/Surgency		Negative Emotionality		Orienting/Regulatory Capacity	
Variable ^1^	*n* (%) or Mean (SD)	Mean (SD) or β (95% CI)	*p*-Value	Mean (SD) or β (95% CI)	*p*-Value	Mean (SD) or β (95% CI)	*p*-Value
Categorical variable							
Maternal education level, *n* (%)							
Did not finish high school	4 (2.5%)	5.92 (0.98)	0.18	4.92 (1.05)	0.22	4.90 (1.10)	0.004 *
High school graduate or GED	23 (14.5%)	5.74 (0.69)		4.30 (1.0)		5.76 (0.53)	
Some college	40 (25.2%)	5.59 (0.67)		4.13 (0.95)		5.49 (0.70)	
College graduate or above	92 (57.9%)	5.46 (0.64)		4.02 (0.96)		5.33 (0.67)	
Race of mother, *n* (%)							
White	126 (80.3%)	5.46 (0.63)	<0.001 *	4.02 (0.94)	0.03 *	5.29 (0.69)	0.002 *
Black	21 (13.2%)	5.82 (0.73)		4.53 (0.88)		5.85 (0.58)	
Other	12 (7.5%)	6.04 (0.70)		4.42 (1.25)		5.66 (0.56)	
Infant sex, *n* (%)							
Male	84 (52.8%)	5.72 (0.64)	<0.001 *	4.14 (0.96)	0.69	5.40 (0.73)	0.77
Female	75 (47.2%)	5.36 (0.65)		4.08 (0.98)		5.37 (0.65)	
Breastfeeding status, *n* (%)							
Exclusive breastfeeding	90 (56.6%)	5.51 (0.67)	0.37	4.17 (0.95)	0.68	5.35 (0.73)	0.10
Partial breastfeeding	26 (16.4%)	5.72 (0.63)		4.03 (0.96)		5.57 (0.76)	
Not breastfeeding	43 (27.0%)	5.53 (0.68)		4.02 (1.02)		5.49 (0.67)	
Continuous variable							
Maternal age (year), mean (SD)	31.4 (5.1)	−0.03 (−0.05, −0.008)	0.006 *	−0.04 (−0.07, −0.01)	0.009 *	−0.006 (−0.03, 0.02)	0.61
Pre-pregnancy BMI, mean (SD)	27.8 (7.2)	0.01 (−0.005, 0.02)	0.18	0.001 (−0.02, 0.02)	0.96	0.01 (−0.002, 0.03)	0.09
Gestational age at delivery (week), mean (SD)	38.8 (1.5)	0.03 (−0.04, 0.10)	0.38	0.02 (−0.08, 0.13)	0.67	−0.05 (−0.13, 0.02)	0.14
Infant age at IBQ measurement (month), mean (SD)	9.4 (0.7)	0.004 (−0.001, 0.008)	0.14	0.04 (−0.003, 0.01)	0.32	0.002 (−0.004, 0.007)	0.55

^1^ Categorical variables are displayed as *n* (%). The difference in temperament scores between categorical variables was analyzed using One-Way Analysis of Variance (ANOVA). Continuous variables are displayed as a mean (SD). The association between continuous variables and temperament scores were analyzed using univariate linear regression. * *p*-value < 0.05.

**Table 2:** Association between alpha diversity and infant temperament ^1^.

	Shannon		Chao 1	
Scale	Beta	*p*-Value	Beta	*p*-Value
Positive affect/surgency	0.11	0.27	0.001	0.63
Negative Emotionality	−0.18	0.24	−0.003	0.12
Orienting/regulatory capacity	0.01	0.92	−0.001	0.62

^1^ Linear regression models were adjusted for maternal age, maternal education, maternal race, maternal pre-pregnancy BMI, gestational age, birth weight, infant sex, and infant age at IBQ-RVSF collection.

**Table 3:** Association between gut microbiota clusters and infant temperament scales ^1^.

Scale	Cluster	Univariate Model			Multivariate Model		
		Beta	*p*-Value	q-Value	Beta	*p*-Value	q-Value ^2^
Positive affect/surgency	Cluster C	ref	-	-	ref	-	-
	Cluster B	0.17 (−0.11, 0.46)	0.22	0.22	0.14 (−0.13, 0.41)	0.30	0.31
	Cluster A	0.41 (0.08, 0.74)	0.01 *	0.03 *	0.29 (−0.03, 0.61)	0.07	0.11
Negative emotionality	Cluster C	ref	-		ref	-	-
	Cluster B	0.44 (0.03, 0.85)	0.04 *	0.12	0.44 (0.02, 0.86)	0.04 *	0.12
	Cluster A	0.57 (0.09, 1.05)	0.02 *	0.03 *	0.55 (0.05, 1.04)	0.03 *	0.09
Orienting/regulatory capacity	Cluster C	ref	-		ref	-	-
	Cluster B	0.20 (−0.11, 0.51)	0.21	0.22	0.16 (−0.15, 0.47)	0.31	0.31
	Cluster A	0.24 (−0.12, 0.61)	0.20	0.20	0.20 (−0.17, 0.57)	0.30	0.30

^1^ Multivariate models were adjusted for maternal education, maternal age, maternal race, pre-pregnancy BMI, gestational age, infant sex, birth weight and infant age at IBQ-RVSF collection. ^2^ *p*-values were adjusted using false discovery rate (FDR) correction for multiple comparisons using the Benjamini–Hochberg procedure. * *p*-value < 0.05.

**Table 4:** Differential abundance testing: associations between gut microbiota taxa at the genus level and positive affect/surgency, adjusted using covariates ^1^.

Phylum	Family	Genus	Q Value for DESeq2 ^2^	Q Value for ANCOMBC	Q Value for MaAslin
Firmicutes	Christensenellaceae	Christensenellaceae R-7 group	6.3 × 10^−7^	0.03	0.11
Firmicutes	Oscillospiraceae	UCG-002	6.3 × 10^−7^	0.03	0.11
Firmicutes	Peptostreptococcaceae	Clostridioides	1.0 × 10^−3^	0.11	0.16
Firmicutes	Ruminococcaceae	Subdoligranulum	8.7 × 10^−4^	0.23	0.22

^1^ Model was adjusted for maternal education, maternal age, maternal race, pre-pregnancy BMI, gestational age, infant sex, birth weight, and infant age at IBQ-RVSF collection. ^2^ Q-value is the FDR-adjusted *p*-value. Q-value < 0.001, 0.25, and 0.25 for multiple comparisons was considered statistically significant for DESeq2, ANCOM-BC, and MaAslin, respectively.

## Data Availability

The data presented in this study are available on request from the corresponding author.

## Appendix A

**Table A1:** Association between gut microbiota clusters and infant temperament scales, which were stratified in accordance with infant vitamin D intake ^1^.

Scale	Cluster	No Vitamin D (59)			Vitamin D (100)		
		Beta	*p*-Value	q-Value ^2^	Beta	*p*-Value	q-Value ^2^
Positive affect/surgency	Cluster A	ref	-	-	ref	-	-
	Cluster B	0.06 (−0.38, 0.50)	0.78	0.84	0.14 (−0.27, 0.56)	0.50	0.50
	Cluster C	0.31 (−0.22, 0.84)	0.24	0.36	0.28 (−0.21, 0.76)	0.26	0.26
Negative Emotionality	Cluster A	ref	-	-	ref	-	-
	Cluster B	0.58 (−0.06, 1.23)	0.08	0.24	0.60 (−0.04, 1.23)	0.06	0.18
	Cluster C	0.94 (0.17, 1.71)	0.02 *	0.06	0.59 (−0.15, 1.33)	0.11	0.26
Orienting/regulatory capacity	Cluster A	ref	-	-	ref	-	-
	Cluster B	0.05 (−0.47, 0.57)	0.84	0.84	0.19 (−0.27, 0.65)	0.42	0.50
	Cluster C	−0.03 (0.64, 0.59)	0.93	0.93	0.37 (−0.16, 0.91)	0.17	0.26

^1^ Models were adjusted for maternal education, maternal age, maternal race, pre-pregnancy BMI, gestational age, infant sex, birth weight, and infant age at IBQ-RVSF collection. ^2^ *p*-values were adjusted using false discovery rate (FDR) correction for multiple comparisons using the Benjamini–Hochberg procedure. * *p*-value < 0.05.

**Table A2:** Association between gut microbiota clusters and infant temperament scales, which were stratified in accordance with infant sex ^1^.

Scale	Cluster	Male (84)			Female (75)		
		Beta	*p*-Value	q-Value ^2^	Beta	*p*-Value	q-Value ^2^
Positive affect/surgency	Cluster A	ref	-	-	ref	-	-
	Cluster B	0.20 (−0.21, 0.62)	0.34	0.34	0.06 (−0.30, 0.43)	0.73	0.73
	Cluster C	0.26 (−0.23, 0.74)	0.30	0.45	0.29 (−0.21, 0.79)	0.25	0.38
Negative Emotionality	Cluster A	ref	-	-	ref	-	-
	Cluster B	0.86 (0.27, 1.46)	0.005 *	0.02 *	0.18 (−0.40, 0.76)	0.53	0.73
	Cluster C	0.92 (0.25, 1.60)	0.008 *	0.02 *	0.52 (−0.27, 1.30)	0.19	0.38
Orienting/regulatory capacity	Cluster A	ref	-	-	ref	-	-
	Cluster B	0.39 (−0.12, 0.90)	0.13	0.20	−0.12 (−0.50, 0.26)	0.54	0.73
	Cluster C	0.20 (−0.38, 0.78)	0.49	0.50	0.08 (−0.44, 0.59)	0.76	0.76

^1^ Models were adjusted for maternal education, maternal age, maternal race, pre-pregnancy BMI, gestational age, birth weight, and infant age at IBQ-RVSF collection. ^2^ *p*-values were adjusted using false discovery rate (FDR) correction for multiple comparisons using the Benjamini–Hochberg procedure. * *p*-value < 0.05.

**Table A3:** Coefficient of differential abundance testing: associations between gut microbiome taxa at genus level and positive affect/surgency, adjusted using covariates ^1^.

Phylum	Family	Genus	DESeq2	ANCOMBC	MaAslin
Firmicutes	Christensenellaceae	R-7 group	1.87	0.39	0.52
Firmicutes	Oscillospiraceae	UCG-002	1.69	0.37	0.52
Firmicutes	Peptostreptococcaceae	Clostridioides	−1.26	−0.44	−0.49
Firmicutes	Ruminococcaceae	Subdoligranulum	1.13	0.38	0.51

^1^ Model was adjusted for maternal education, maternal age, maternal race, pre-pregnancy BMI, gestational age, infant sex, birth weight, and infant age at IBQ-RVSF collection.
